# Draft Whole-Genome Sequence of a Uropathogenic Escherichia coli Strain Carrying the *eae* Gene

**DOI:** 10.1128/MRA.00980-19

**Published:** 2019-10-24

**Authors:** Tiago Barcelos Valiatti, Fernanda Fernandes Santos, Ana Carolina de Mello Santos, Rosa Maria Silva, Eneas Carvalho, Tânia Aparecida Tardelli Gomes

**Affiliations:** a Departamento de Microbiologia, Imunologia e Parasitologia, Escola Paulista de Medicina, Universidade Federal de São Paulo, São Paulo, Brazil; b Laboratório de Bacteriologia, Instituto Butantan, São Paulo, Brazil; Indiana University, Bloomington

## Abstract

Uropathogenic Escherichia coli (UPEC) strains are responsible for most cases of urinary tract infections worldwide. We present the draft whole-genome sequence of the UPEC 252 strain, which carries the *eae* gene that encodes the intimin adhesin. Intimin promotes intimate adherence of enteropathogenic E. coli and enterohemorrhagic E. coli to intestinal cells.

## ANNOUNCEMENT

Urinary tract infection (UTI) is considered one of the most frequent infections affecting the community and hospitalized individuals worldwide (1). The main causative agent of UTI is uropathogenic Escherichia coli (UPEC), which represents about 75 to 90% of cases (1–3).

This announcement aims to describe a draft whole-genome sequence of UPEC 252, isolated in 1999 from a 1-month-old female patient with a UTI who was admitted to Hospital São Paulo, a public hospital in São Paulo, Brazil. The UPEC 252 strain was originally characterized as a carrier of the *eae* gene (4), which encodes intimin, the main adhesin involved in the ability of enteropathogenic E. coli (EPEC) and enterohemorrhagic E. coli (EHEC) to promote attaching and effacing (AE) lesions in epithelial cells (5).

UPEC 252 was stored in lysogeny broth (LB) plus 15% glycerol and kept at –80°C. One isolated colony obtained on MacConkey agar was cultured overnight in LB at 37°C, and DNA extraction was performed using the Wizard genomic DNA purification kit (Promega USA), following the manufacturer’s recommendations. Genomic DNA was fragmented by tagmentation using the Nextera XT DNA library preparation kit, according to the manufacturer’s instructions, yielding fragments with an average size of 1 kb. A genomic library was prepared using the HiSeq rapid SBS v2 library kit (Illumina USA), which was subsequently sequenced on the Illumina HiSeq 1500 platform using the 2 × 250-bp protocol. The total number of reads sequenced was 5,267,541, which was reduced to 4,876,002 reads (92.5%) after the removal of low-quality bases (regions with a mean quality score below 25 were discarded), small reads (shorter than 40 bases), and Illumina adaptors using the software Trimmomatic (6). The data that originated from the Illumina readings were assembled using SPAdes version 3.12.0 (7), annotation was performed using Prokka version 1.13.3 (8), and the genome assembly metric was calculated using QUAST. All software programs were used with the default settings.

The genome of UPEC 252 presented a total size of 4,802,947 bp (GC content, 50.46%) distributed in 151 contigs, the largest being 322,751 bp. Further, the *N*_50_ and *N*_75_ values were 187,460 and 87,691 bp, respectively, while the *L*_50_ and *L*_75_ values were 10 and 20 contigs, respectively. The annotation showed that UPEC 252 has 1 transfer-messenger RNA (tmRNA) gene, 83 tRNA genes, 8 rRNA genes, and 4,520 coding sequences (CDS).

The contigs were submitted to the VirulenceFinder software (version 2.0), Center for Genomic Epidemiology (9), to identify the genes belonging to the pathogenicity island of approximately 35 kb called the locus of enterocyte effacement (LEE). The LEE includes the *eae* gene, which is required for AE lesion formation, suggesting that UPEC 252 has hybrid potential, containing virulence genes associated with the establishment of diarrhea and urinary tract infections.

We expect that knowledge of the UPEC 252 genome will contribute to the support of future studies on the true virulence potential of hybrid pathogenic E. coli strains.

### Data availability.

The reads used for assembly of the UPEC 252 genome were deposited in the Sequence Read Archive (SRA) at the NCBI under the accession number SRR9317828, and the whole-genome shotgun sequences were deposited in the GenBank database under the accession number VFST00000000. The versions described in this paper are the first versions.

## References

[1] Hooton T 2012 Uncomplicated urinary tract infection. N Engl J Med 366:1028–1037. doi:10.1056/NEJMcp1104429. 22417256

[2] Korbel L, Howell M, Spencer JD 2017 The clinical diagnosis and management of urinary tract infections in children and adolescents. Paediatr Int Child Health 37:273–279. doi:10.1080/20469047.2017.1382046. 28978286

[3] Ronald A 2003 The etiology of urinary tract infection: traditional and emerging pathogens. Dis Mon 49:71–82. doi:10.1067/mda.2003.8. 12601338

[4] Abe CM, Salvador FA, Falsetti IN, Vieira MA, Blanco J, Blanco JE, Blanco M, Machado AM, Elias WP, Hernandes RT, Gomes TA 2008 Uropathogenic *Escherichia coli* (UPEC) strains may carry virulence properties of diarrhoeagenic *E. coli*. FEMS Immunol Med Microbiol 52:397–406. doi:10.1111/j.1574-695X.2008.00388.x. 18336383

[5] Kaper JB, Nataro JP, Mobley HL 2004 Pathogenic *Escherichia coli*. Nat Rev Microbiol 2:123–140. doi:10.1038/nrmicro818. 15040260

[6] Bolger AM, Lohse M, Usadel B 2014 Trimmomatic: a flexible trimmer for Illumina sequence data. Bioinformatics 30:2114–2120. doi:10.1093/bioinformatics/btu170. 24695404 PMC4103590

[7] Bankevich A, Nurk S, Antipov D, Gurevich AA, Dvorkin M, Kulikov AS, Lesin VM, Nikolenko SI, Pham S, Prjibelski AD, Pyshkin AV, Sirotkin AV, Vyahhi N, Tesler G, Alekseyev MA, Pevzner PA 2012 SPAdes: a new genome assembly algorithm and its applications to single-cell sequencing. J Comput Biol 19:455–477. doi:10.1089/cmb.2012.0021. 22506599 PMC3342519

[8] Seemann T 2014 Prokka: rapid prokaryotic genome annotation. Bioinformatics 30:2068–2069. doi:10.1093/bioinformatics/btu153. 24642063

[9] Joensen KG, Scheutz F, Lund O, Hasman H, Kaas RS, Nielsen EM, Aarestrup FM 2014 Real-time whole-genome sequencing for routine typing, surveillance, and outbreak detection of verotoxigenic *Escherichia coli*. J Clin Microbiol 52:1501–1510. doi:10.1128/JCM.03617-13. 24574290 PMC3993690

